# DiscoVerse: multi-agent pharmaceutical co-scientist for traceable drug discovery and reverse translation

**DOI:** 10.3389/frai.2026.1808378

**Published:** 2026-06-18

**Authors:** Xiaochen Zheng, Alvaro Serra, Ilya Schneider Chernov, Maddalena Marchesi, Eunice Musvasva, Tatyana Y. Doktorova

**Affiliations:** 1 Predictive Modelling, F. Hoffmann-La Roche Ltd., Basel, Switzerland; 2 Clinical Safety, F. Hoffmann-La Roche Ltd., Basel, Switzerland; 3 Translational Safety, F. Hoffmann-La Roche Ltd., Basel, Switzerland

**Keywords:** drug discovery, knowledge retrieval, large language models, multi-agent systems, reverse translation

## Abstract

Pharmaceutical research and development has accumulated vast and heterogeneous archives of data. Much of this knowledge stems from discontinued programs, and reusing these archives is invaluable for reverse translation. However, in practice, such reuse is often infeasible. In this work, we introduce *DiscoVerse*, a multi-agent co-scientist designed to support pharmaceutical research and development at Roche. Designed as a human-in-the-loop assistant, *DiscoVerse* enables domain-specific queries by delivering evidence-based answers: it retrieves relevant data, links across documents, summarizes key findings and preserves institutional memory. We assess *DiscoVerse* through expert evaluation of source-linked outputs. Our evaluation spans a selected subset of 180 molecules from Roche's research and development repositories, encompassing over 0.87 billion Byte-Pair Encoding (BPE) tokens and more than four decades of research. To our knowledge, this represents the first agentic framework to be systematically assessed on real pharmaceutical data for reverse translation, enabled by authorized access to confidential archives covering the full lifecycle of drug development. Our contributions include: role-specialized agent designs aligned with scientist workflows; human-in-the-loop support for reverse translation; expert evaluation; and a large-scale demonstration showing promising decision-making insights. In brief, across seven benchmark questions, *DiscoVerse* achieved near-perfect recall (≥0.99) with moderate precision (0.71 − 0.91). Qualitative assessments and three real-world pharmaceutical use cases further showed faithful, source-linked synthesis across preclinical and clinical evidence.

## 1 Introduction

The pharmaceutical industry has generated large volumes of experimental data over decades. For each drug candidate, companies typically produce internal study reports, raw experimental data, toxicological findings, histopathological evaluations, nonclinical and clinical study presentations, and documentation of decision-making processes across drug discovery and development. Together, these materials form a large but under used resource.

A substantial portion of these data originates from discontinued drug candidates halted at different stages of development. Although attrition is inherent to pharmaceutical research, these data remain valuable. Embedded within them are treatment-related findings, target organ toxicities, safety and efficacy signals, and detailed experimental methods. Through **reverse translation** (Kasichayanula and Venkatakrishnan, 2018; Honkala et al., 2022; Vanmeerbeek et al., 2023; Sokol et al., 2025), clinical outcomes can inform earlier-stage research, enabling identification of new targets and biomarkers (Shakhnovich, 2018) and reuse of lessons from clinical pharmacology and regulatory review (Faucette et al., 2018). A representative example is the discovery of the TGF-β pathway as a therapeutic target (Mariathasan et al., 2018) by tracing treatment resistance observed in clinical studies back to underlying biology.

A key challenge in reverse translation is scale and fragmentation. End-to-end drug development produces thousands of heterogeneous documents distributed across organizations and external partners, with inconsistent naming conventions and evolving project-specific terminology. As a result, manual browsing and keyword-based search are ineffective. Synonymy, terminology drift, tabular data, and context-dependent phrasing lead to missed evidence and spurious matches. Addressing this requires **semantic retrieval**, **cross-document linking**, **preclinical–clinical data alignment**, and **auditable synthesis** (Honkala et al., 2022; Henrique Vieira E Vieira et al., 2025). When curated and made searchable, legacy datasets can support quantitative analysis, machine learning, and predictive toxicology across programs, reducing redundant experimentation and improving decision-making.

Recent large language models (LLMs) (Achiam et al., 2023; Team et al., 2023; Anthropic, 2024; Bai et al., 2023; Liu et al., 2024; Guo et al., 2025) can read and synthesize large unstructured corpora and generate concise, source-grounded summaries of biomedical text (Tung et al., 2024; Van Veen et al., 2024; Williams et al., 2025; Ghim and Ahn, 2023; Wornow et al., 2025; Gallifant et al., 2025; Lai et al., 2024). These capabilities make LLMs a practical tool for applying consistent criteria across many historical reports, allowing relevant evidence to surface at scale.

However, pharmaceutical research and development poses challenges for single-agent LLMs. Studies in large pharmaceutical industries are highly context-specific, with differences in endpoints, assays, and terminology that limit the effectiveness of standardized prompts and instructions for in-context learning (Brown et al., 2020; Wei et al., 2022a,b). In addition, collaboration with multiple contract research organizations introduces further heterogeneity. As a result, single-agent systems often fail to generalize from sparse or non-representative information sources (Reizinger et al., 2024; Sengupta et al., 2025; Peters and Chin-Yee, 2025). These issues are compounded by siloed data and long-term terminology drift. Furthermore, studies are organized on a per-molecule basis, and throughout the entire drug development lifecycle, the accumulated documentation for each molecule far exceeds the context window of modern LLMs.

We therefore adopt a multi-agent system (MAS) (Boiko et al., 2023; Hong et al., 2023; Wu et al., 2024; Kapoor et al., 2024; Wang Z. et al., 2025; Gottweis et al., 2025; Zhu et al., 2025b) architecture with role-specialized agents for retrieval, research, verification, and synthesis. By enabling agents to critique one another and enforcing source-grounded outputs, MAS supports traceable and auditable reasoning, which is essential in regulated pharmaceutical research. Prior work shows that MAS can outperform single-agent systems in specialized domains with limited demonstrations (Wang et al., 2024; Chen et al., 2025; Wang Z. et al., 2025; Jin et al., 2025; Zhu et al., 2025b; Wu et al., 2024; Huang et al., 2025; Zhu et al., 2025a; Seal et al., 2025).

Building on this framework, we developed *DiscoVerse*, a multi-agent co-scientist designed for pharmaceutical knowledge ingestion and translational analysis across the drug development lifecycle. *DiscoVerse* integrates preclinical and clinical data to support systematic mapping between experimental findings and clinical outcomes. Operating as a human-in-the-loop system, it allows scientists to query historical programs (e.g., reasons for compound discontinuation) and retrieves linked molecular, toxicological, and clinical evidence. In doing so, *DiscoVerse* supports reverse translation and reuse of past development experience in current research programs.

The key contributions of our framework are four-fold: (i) **specialized agent designs** that mirror scientist workflows, each optimized for a distinct sub-task with external knowledge available; (ii) integration with **human-in-the-loop into the drug-design workflow**, moving from long-document ingestion through domain-specific question-answering and comparative historical analysis to actionable design insights and reverse translation; (iii) **expert evaluation on real-world pharmaceutical data**: unlike prior work that relies primarily on curated benchmark datasets, we build and systematically evaluate our framework using authentic pharmaceutical research and development data; and (iv) **large-scale demonstration** on a real pharmaceutical knowledge repository, showing promising results in answer accuracy and decision-context insights that were previously time-consuming to assemble.

**Relation to existing AI-driven drug discovery systems** Recent work has produced several multi-agent LLM systems for pharmaceutical and biomedical research, each targeting different stages of the discovery pipeline. The AI Co-Scientist (Gottweis et al., 2025) employs a “generate, debate, and evolve” paradigm over public literature to produce novel hypotheses for drug repurposing and target identification. PharmaAgents (Gao et al., 2025) simulates a virtual pharma pipeline for small-molecule design, from target identification through lead optimization. TxGemma (Wang E. et al., 2025), BioDiscoveryAgent (Roohani et al., 2025), and BioReason (Fallahpour et al., 2026) focus on molecular property prediction and genetic perturbation experiment design, respectively, leveraging specialized toolchains for computational biology. BioMNI (Huang et al., 2025) provides a general-purpose biomedical agent combining LLM reasoning with dynamic tool execution across public databases. At the workflow level, (Shahin et al. 2025) propose AI agents for clinical pharmacology and translational science tasks. A comprehensive survey by (Seal et al. 2025) categorizes these systems across ReAct, Reflection, Supervisor, and Swarm architectures.

*DiscoVerse* differs from these systems in three respects. First, it operates over proprietary, confidential pharmaceutical archives rather than public literature or molecular databases, requiring robust handling of heterogeneous legacy documents with inconsistent formats. Second, it explicitly targets *reverse translation*, which links clinical outcomes back to preclinical evidence across discontinued programs. This is a use case which has not been addressed by existing systems. Third, it enforces end-to-end source traceability through structured schemas, designed for deployment in regulated environments where auditability is a hard requirement rather than a desirable feature. These distinctions position *DiscoVerse* as complementary to hypothesis-generation and molecular-design agents: where those systems propose new candidates, *DiscoVerse* mines institutional memory to inform whether and how such candidates should advance.

## 2 Methodology

**Figure 1:**
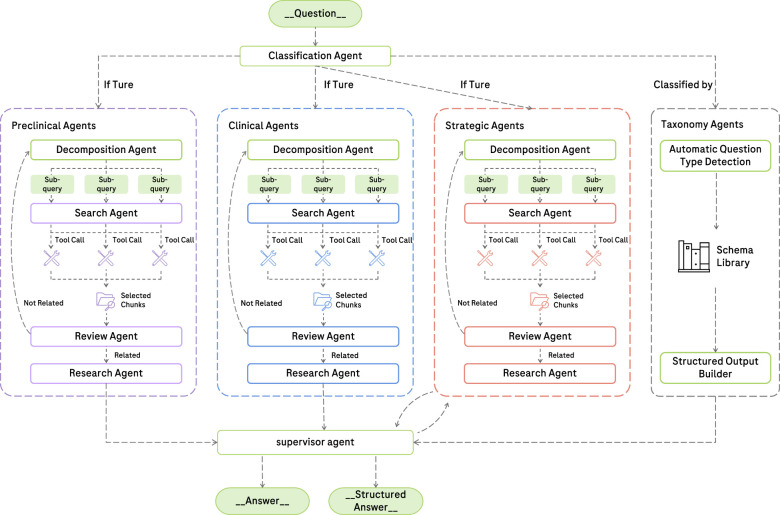
The illustrative overview of *DiscoVerse*. The Discoverse orchestrates three specialized **Preclinical**, **Clinical**, and **Strategic** branches. Within each branch, **Decomposition Agent** follows a read–rewrite–retrieve (Ma et al., 2023) workflow by generating sub-queries, calling tools to access the DiscoVerse database, and retrieving relavant chunks. If gaps remain, **Review Agent** will return to decomposition agent for refinement. A **Research Agent** discovers and synthesizes scientific findings, while the **Supervise Agent** orchestrates the process, deciding which branch outputs to integrate. Finally, a **Taxonomy Agent** produces structured answers through an expert-in-the-loop schema library for consistent answer output.

*DiscoVerse* is implemented as a modular multi-agent system with retrieval tool calling, as illustrated in Figure 1. The system consists of role-specialized agents that mirror common scientific workflows in pharmaceutical research and development:

1. **Preclinical agents** address questions related to research conducted before first-in-human studies, including *in vitro* assays and animal toxicology studies.
2. **Clinical agents** focus on research activities after entry into human studies, such as clinical trials and clinical safety evaluations.
3. **Strategic agents** handle higher-level questions, including portfolio decisions, program discontinuation rationales, and cross-project comparisons.

These domain agents are coordinated by a **Supervisor Agent**, which orchestrates query routing, tracks execution, and integrates results across domains. This design allows each agent to operate with domain-specific prompts and logic while ensuring that findings are combined coherently. All agents communicate through structured messages, and the Supervisor ensures that outputs remain aligned with the original user query.

To support consistent and auditable outputs (Karmaker Santu and Feng, 2023; Dagdelen et al., 2024; Zaratiana et al., 2025), *DiscoVerse* also includes **Taxonomy Agents**. These agents operate downstream of the domain agents and map extracted evidence into a schema library co-developed with scientists and project leads. The schema library encodes expert-defined question types, including classification rules, required evidence elements, and structured output templates. A single user query may map to multiple question types, enabling composition across preclinical, clinical, and strategic dimensions while preserving provenance.

When a user submits a query to *DiscoVerse*, it is processed through a coordinated multi-agent workflow that includes query classification, query decomposition (Ma et al., 2023), document retrieval, relevance filtering, domain-specific evidence extraction, cross-agent synthesis, and structured output generation. Each step is handled by a specialized agent to reduce prompt complexity and improve interpretability.

At a high level, *DiscoVerse* decomposes complex questions into smaller, agent-managed tasks. The input queries are first classified and decomposed into domain-specific sub-queries, which are routed to the appropriate agents. Relevant documents are then retrieved using a hybrid symbolic–semantic search approach, reviewed for relevance, and distilled into domain-specific findings. The Supervisor Agent integrates these findings into a unified response, while Taxonomy Agents map the results into structured schemas to support consistency and auditability.

The full end-to-end workflow, including agent interactions, retrieval strategies, relevance thresholds, and fallback mechanisms, is described in detail in Appendix A.

## 3 Materials

Our documentation repository contains 872,453,585 BPE (Sennrich et al., 2016) tokens^1^ in 15,762 PDF files covering 180 selected drug molecules. On average, each molecule is associated with approximately 10,000 pages of documentation, forming a comprehensive collection that includes animal study reports, clinical study reports, meeting minutes, project summary presentations, and investigator brochures. A substantial fraction of the data originates from pre-clinical *in vivo* animal studies.

The dataset spans a period of more than four decades. Consequently, the collection includes scanned copies of legacy documents, exhibiting considerable variability in linguistic style, level of technical detail, and structural format. This heterogeneity, particularly the presence of non-digitally born documents, presents a substantial challenge for reliable text retrieval, parsing, and data harmonization. The detailed data preparation pipeline (including embedding and indexing) is described in Appendix B.

## 4 Benchmark questions

**Figure 2:**
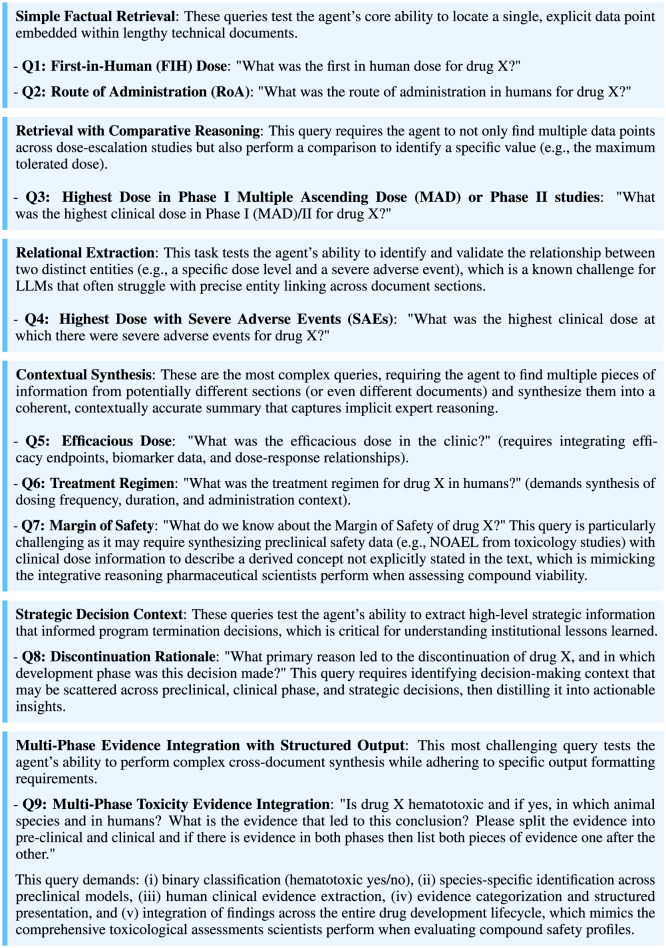
The design and explanation of benchmark questions.

To ensure our evaluation reflects authentic scientist needs and comprehensively tests *DiscoVerse*'s capabilities, we collaborated actively with toxicology project leaders and safety clinical and pre-clinical scientists to identify the types of questions routinely asked when evaluating discontinued programs. Therefore, as shown in Figure 2, we designed nine **preclinical and clinically relevant questions spanning the entire drug development lifecycle**, each intended to probe a distinct facet of information extraction relevant to late-stage drug development. These questions were derived from real decision-making scenarios and needs: assessing whether a shelved compound could be repurposed, understanding why similar molecular scaffolds failed previously, or determining the optimal upper dose range for new candidate dose-range-finding studies using historical data and precedent analyses explaining prior dosing decisions. Collectively, these queries represent a spectrum of complexity, ranging from simple factual retrieval to complex synthesis of disparate information. Meanwhile, the design strategy ensures that our evaluation is both **scientifically grounded** (reflecting questions scientists actually need answered) and **comprehensively diagnostic** (systematically testing each capability required for effective pharmaceutical knowledge extraction).

## 5 Results

### 5.1 Experimental setup

Our experiments aim to test and assess the system-level performance of *DiscoVerse*'s multi-agent architecture rather than benchmarking individual LLM capabilities. All agents were powered by GPT-4.1 (gpt-4.1-2025-04-14) to isolate architectural contributions from model-specific variations. We run the embedding and reranker model locally on NVIDIA A100 GPU.

We executed *DiscoVerse* for each query (Q1–Q9) on each molecule in our dataset, generating both unstructured (natural language) and structured (formatted) outputs for each molecule-query pair. To enable rigorous expert evaluation and ensure traceability, we logged all retrieved document chunks, intermediate agent outputs, and source attributions.

### 5.2 Results for benchmark questions

We evaluated *DiscoVerse* across seven benchmark questions designed to simulate real pharmaceutical information retrieval tasks. Complete metrics (accuracy, precision, recall, specificity, and F1-score) appear in the Table 1. The design for expert evaluation is described in Appendix C, and the detail criteria for each benchmark question is shown in Appendix D. We choose accuracy, precision, recall, specificity and F1-score to quantify the performance, as described in Appendix E. The results show a consistent performance pattern that defines how the system should be used in drug development workflows.

**Table 1:** Per-query performance of *DiscoVerse*.

Query	Accuracy	Precision	Recall	Specificity	F1-score
Q1 (FIH dose)	0.9086	0.8712	1.0000	0.7605	0.9311
Q2 (Route of administration)	0.8852	0.8444	1.0000	0.6956	0.9156
Q3 (Highest phase I/II dose)	0.8524	0.7890	1.0000	0.6707	0.8820
Q4 (Highest dose with SAEs)	0.9243	0.8488	0.9864	0.8828	0.9125
Q5 (Efficacious dose)	0.8857	0.7142	1.0000	0.8400	0.8333
Q6 (Treatment regimen)	0.9426	0.9078	1.0000	0.8679	0.9517
Q7 (Margin of safety)	0.8677	0.8139	1.0000	0.6862	0.8974

Across queries, *DiscoVerse* achieves near-perfect recall (1.0000 for six queries; 0.9864 for Q4), ensuring that relevant items are rarely missed. Where performance varies is in precision and specificity. Precision ranges from 0.7142 (Q5) to 0.9078 (Q6), indicating a tendency toward false positives. Complementing this view, specificity spans 0.6707 (Q3) to 0.8828 (Q4), showing how often the system correctly rejects non-matching content. In other words, when the agent admits borderline or context-mismatched items, both precision (positive-set purity) and specificity (negative-set rejection) decline. Lower specificity reflects many negatives being incorrectly marked as positives. Together with the precision range above, this paints a consistent picture: the system aggressively retrieves candidates (high recall) but sometimes over-includes near-miss evidence, lowering both precision and specificity.

Expert evaluation indicates the false positives are not hallucinations but failures of contextual understanding. Each error class simultaneously reduces precision (adds incorrect items to the positive set) and specificity (converts true negatives into false positives): the agent might extract a preclinical dose when asked for a clinical one (described in Table 2 for Q1 and Table 2 for Q3), describe planned as an actual administration route (described in Table 2 for Q1 and Table 2 for Q2), confuse trial phases (described in Tab. Table 2 for Q3), link a dose to an unrelated adverse event (described in Table 2 for Q4), report efficacy for doses planned but not yet tested in humans (described in Table 2 for Q5), provide incorrect dose-specific details like wrong infusion durations (described in Tab. Table 2 for Q6), or conflate preclinical safety margins with clinical data (described in Table 2 for Q7)

**Table 2:** Error analysis - examples of false positives and correct samples.

Correctness	Agent answer	True answer	Error type
Q1 (FIH does)			
✗ - FP	The first-in-human dose for X was 100 mg/kg administered orally (by gavage) to cynomolgus monkeys in a single ascending dose study.	No planned dose in available data. Only first dosing available for monkeys.	Preclinical and clinical confusion
✗ - FP	The first-in-human (FIH) dose for X was 0.75 mg/kg administered as a single oral dose to healthy volunteers.	The planned FIH was 0.75 mg/kg but has not yet been tested in humans.	Planned and actual confusion
✓ - TP	The first-in-human dose for X was 400 mg as a single oral dose, administered to healthy Asian male subjects in Cohort 1 of the Phase I SAD study Y		—
Q2 (Route of administration)			
✗ - FP	The route of administration in humans for drug X was oral (by gavage).	The dose was not yet tested in humans. The route was planned to be oral but not yet tested.	Planned and actual confusion
✓ - TN	There is no evidence of X being administered to humans in any clinical trial according to the provided information. The oral route is repeatedly stated as the intended route but this is based solely on preclinical rationale.		—
✓ - TP	In all human clinical trials, X was administered exclusively via the intranasal route using a metered-dose nasal spray device. No other routes of administration were used in humans		—
Q3 (Highest phase I/II dose)			
✗ - FP	The highest clinical dose administered in Phase I (Multiple Ascending Dose) studies of X was 1.29 × 10^12^ vector genomes (vg) per eye, delivered as a single intravitreal injection of 100 μL per eye. No Phase II clinical dose data are available.	No MAD studies were performed in humans. This dose was from a pre-clinical MAD study.	Preclinical and clinical confusion
✗ - FP	The highest clinical dose administered in Phase I (Multiple Ascending Dose) trials for X was 40 mg as an intravenous (IV) infusion over 48 hours. Phase II studies had not been initiated or reported at the time of the latest available data.	The highest clinical dose (40 mg) was administered in Phase I Single Ascending Dose (SAD) trials, not Multiple Ascending Dose (MAD) trials.	SAD and MAD confusion
15.6-7.5,-26.3499pt ✓ - TP	The highest clinical dose of X administered in Phase I (MAD) trials was 35 mg once weekly (QW) intravenously, achieved in escalation cohorts (e.g., 20/20/35-mg). In Phase II trials, the highest dose tested was 15 mg QW or Q2W (in combination with drug Y), with most studies using 10 mg as the recommended dose for further development.		—
Q4 (Highest dose with SAEs)			
✗ - FP	The highest clinical dose at which SAEs were observed for X was 250 μg/kg administered intravenously. At this dose, SAEs included capillary leak syndrome (CLS, Grade 2, and Grade 4, the latter being life-threatening and not resolved at reporting), dyspnea (Grade 2), infusion-related reactions (Grade 2), and pyrexia (Grade 1/2, with one case requiring hospitalization).	At the 250 μg/kg dose, reported Grade 3+ SAEs included neutropenia, fatigue, peripheral neuropathy, thrombocytopenia, and anemia, which are not mentioned in the answer.	Incomplete SAE reporting
✗ - FN	The highest clinical dose (SAEs, CTCAE Grade ≥3) were observed for X was 45 mg/kg once weekly (QW). However, none of these SAEs were considered related to X by the investigators, and no dose-limiting toxicities (DLTs) or drug-related SAEs were observed at any dose level.	At the 45 mg/kg dose, relevant SAEs like cachexia and pericardial effusion were reported but missed by the agent.	Missed relevant SAEs
✓ - TP	The highest clinical dose at which severe adverse events (AEs) were reported for X was 2,000 mg as a single oral dose in healthy volunteers. Severe AEs (flu-like symptoms: headache, pyrexia, nausea, myalgia, dizziness, malaise) occurred only at the 1,600 mg and 2,000 mg dose levels. These events were transient, managed with analgesics, and resolved within 1–2 days.		—
Q5 (Efficacious dose)			
✗ - FP	The highest dose of X tested in the clinic was 0.25 mg/kg IV. At this dose, stable disease was observed in 3 of 12 patients (25%). Dose-limiting toxicities (capillary leak syndrome) occurred at 0.25 mg/kg, preventing further escalation.	Hallucination - capillary leak syndrome was not mentioned in the report.	Hallucinated adverse event
✗ - FP	The efficacious dose of X in the clinic is 200–400 mg BID or 600–1,000 mg QD. These doses demonstrated robust antiviral activity with median HBV DNA declines of X log_10_ IU/mL after Y days.	Efficacy in humans has not yet been tested.	Preclinical and clinical confusion
✓ - TP	The efficacious dose of X identified in clinical studies is 250 mg once weekly in combination with Y. This dose was selected as the recommended Phase 2 dose based on integrated PK, PD, safety, and efficacy data from Phase 1/2 trials.		—
Q6 (Treatment regimen)			
✗ - FP	X was administered as an IV infusion on Days 1, 3, and 5 of each 21-day cycle. Each infusion lasted 30 minutes, and treatment continued until disease progression or unacceptable toxicity.	Low dose infusion was 5 min. 30 min was for control or high dose only.	Incorrect dose-specific details
✗ - FP	X was administered as IV infusion with weekly dosing at 20, 30, or 45 mg/kg. Infusion durations were reduced from 90 min for the first dose to 60 or 30 min for subsequent doses if tolerated.	Almost correct. Weekly dosing was up to 60 mg/kg, not 45 mg/kg.	Incorrect dose range
✓ - TP	X was administered orally as immediate-release capsules. Regimens included single ascending doses (200–1,600 mg), every other day dosing (50–2,000 mg), and once weekly dosing (800/1,200 mg). All regimens were observer- or double-blind with matching placebo controls.		—
Q7 (Margin of safety)			
✗ - FP	The Margin of Safety for drug X is considered adequate. In cynomolgus monkeys, the NOAEL (50 mg/kg/day) provides a 5.5-fold (AUC) and 6.2-fold (Cmax) margin over human exposure. In rats, margins are below unity (AUC margin < 1x, Cmax 1x).	NOAEL in rats was 150 mg/kg with margins of 16x and 97x to human Cmax, not below unity.	Incorrect margin calculations
✗ - FP	The Margin of Safety for X is based on preclinical studies and initial Phase 1 clinical studies in healthy volunteers. In clinical studies, no dose-limiting toxicities were observed up to 100 mg single dose and 50 mg multiple dose.	X was not tested in humans yet. All data referenced is preclinical only.	Preclinical and clinical confusion
✓ - TP	The Margin of Safety for X is established based on preclinical studies in cynomolgus monkeys and rats. The NOAEL in monkeys is 0.5 mg/kg/dose IV every 3 weeks, with adverse effects observed at ≥1.4 mg/kg/dose. No human clinical data are available in the first IB.		—

The performance profile with high recall and moderate precision suggests *DiscoVerse* works best as an augmentation tool rather than a standalone system. It automates the most tedious part of knowledge work (comprehensive search) while leaving contextual verification to domain experts. This shifts the workflow from manual “search and synthesize” to AI-assisted “review and discover.” The detailed analysis of Q8 and Q9 is described in Appendix F.

### 5.3 Real-world pharmaceutical industry use cases

#### 5.3.1 Use case 1: reverse translation of hepatotoxicity across species

A common challenge in pharmaceutical research is understanding how clinically observed hepatotoxicity maps back to preclinical models, particularly when findings are weak, inconsistent, or confounded by species limitations. Across modalities including T-cell engagers, immune cytokine-based therapies, antisense molecules, and small molecules, clinically relevant liver injury may emerge despite minimal or ambiguous preclinical signals, complicating dose escalation, and risk assessment.

*DiscoVerse* supports systematic reverse translation by starting from a clinical liver safety signal (e.g., LFT elevations, Hy's law events, hepatocellular injury) and retrieving aligned preclinical, *in vitro*, and mechanistic evidence across species. Applying *DiscoVerse* to discontinued and deprioritized programs identified multiple cases in which clinical hepatotoxicity could be retrospectively examined against preclinical data.

**Figure 3:**
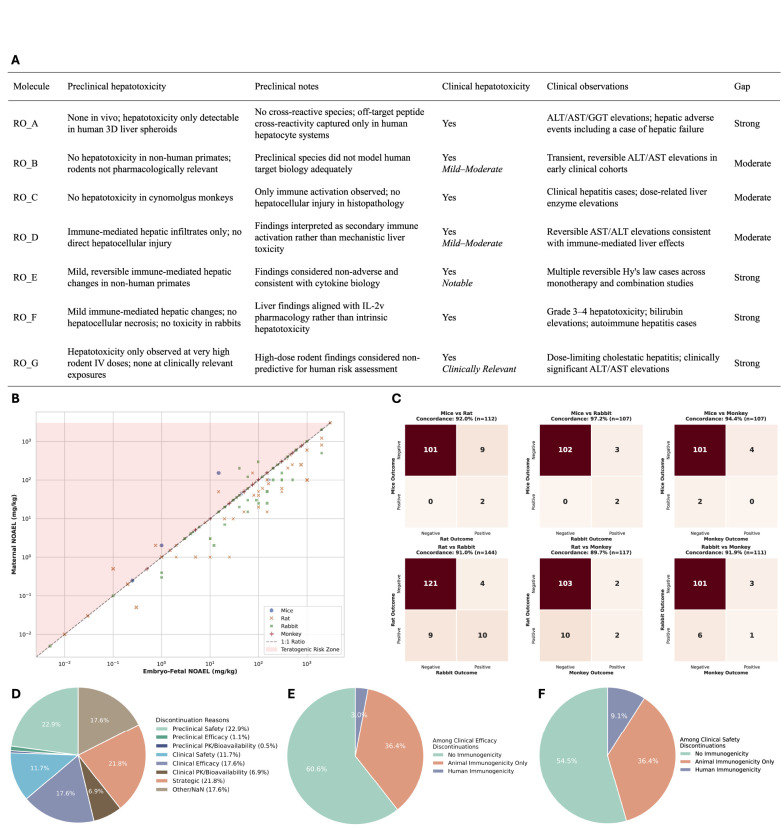
The results of reverse translation, quantitative safety assessment, and immunogenicity insights enabled by *DiscoVerse*; **(A)** reverse translation of hepatotoxicity across species; **(B)** quantitative risk assessment for maternal and embryo-fetal NOAEL; **(C)** species concordance analysis for teratogenicity; retrospective analysis of immunogenicity in discontinued molecule programs, **(D)** discontinuation reasons; **(E)** clinical efficacy by immunogenicity; **(F)** clinical safety by immunogenicity.

As summarized in Figure 3A, several recurring patterns emerged. First, **species non-relevance** was a major contributor to missed signals, particularly for LM modalities (e.g., RO_A and RO_B), where no available species adequately captured either on-target biology or off-target peptide–MHC interactions. Second, **immune-mediated liver effects** were often mild or inconsistent in non-human primates but manifested clinically for molecules such as RO_C, RO_E, and RO_F. Integration of immune activation signals across reports made these links clearer than individual study reviews. Third, **rodent findings were sometimes misleading**, as hepatotoxicity appeared only at supratherapeutic exposures (e.g., RO_G), masking clinical risk. Finally, **human** ***in vitro* systems** provided early indicators of risk in cases such as RO_A, where 3D hepatocyte spheroids identified liability not detected *in vivo*.

Together, this use case shows how *DiscoVerse* strengthens reverse translation by making species gaps, mechanistic signals, and exposure limitations explicit. By converting fragmented archival data into structured, auditable evidence, the system supports earlier recognition of translational risk, informs modality-specific safety strategy, and enables more consistent decision-making across discovery and early development.

#### 5.3.2 Use case 2: quantitative risk assessment in embryo-fetal development

Distinguishing direct embryo-fetal toxicity from effects secondary to maternal toxicity remains a key challenge in developmental safety assessment. This distinction depends on consistent comparison of maternal and embryo-fetal No-Observed-Adverse-Effect Levels (NOAELs), yet such data are typically dispersed across unstructured reports. To quantify relative sensitivity, we defined:


$$\begin{array}{l} \text{Relative Sensitivity Ratio}=\frac{\text{Maternal NOAEL}}{\text{Embryo-Fetal NOAEL}} \end{array}$$ (1)

Using *DiscoVerse*, we extracted paired maternal and embryo-fetal NOAELs, along with *C*_*max*_ and AUC values, across 180 molecules and multiple species. Preclinical Agents validated extracted values by triangulating numeric tables with narrative descriptions, filtering out non-specific toxicity. This simultaneous extraction was of significant interest to project toxicologists, as it enabled the visualization of a specific teratogenicity risk area across the portfolio—an analysis previously infeasible to perform at scale.

As shown in Figure 3B, compounds clustered into distinct risk zones. A “Red Zone” (Relative Sensitivity Ratio >1) identified molecules with embryo-fetal toxicity at exposures below the maternal NOAEL, indicating likely direct teratogenic liability. These patterns allow safety teams to identify legacy risks and apply mechanistic insight to emerging scaffolds.

To assess cross-species consistency, *DiscoVerse* also extracted binary developmental toxicity outcomes across mice, rats, rabbits, and cynomolgus monkeys. As illustrated in Figure 3C, this enabled direct calculation of species concordance rates without manual cross-referencing. The resulting dataset supports quantitative evaluation of model predictivity and more informed selection of relevant species early in development.

#### 5.3.3 Use case 3: strategic retrospective on immunogenicity and attrition

Immunogenicity is frequently observed in preclinical studies, particularly for large molecules, but determining its presence in discontinued clinical programs is challenging when it is not the primary attrition driver. The immunogenicity team sought to identify specific instances of human immunogenicity that might be obscured within programs discontinued for other reasons. The objective was not to re-adjudicate the cause of attrition, but to systematically locate “hidden” positive cases where immunogenicity co-occurred with the primary drivers of clinical safety or clinical efficacy.

Using *DiscoVerse*, discontinued programs were first stratified by primary attrition driver. As shown in Figure 3D, Clinical Safety (11.7%) and Clinical Efficacy (17.6%) were identified alongside Strategic and Preclinical Safety drivers. We then performed focused sub-analyses on the Clinical Efficacy (Figure 3E) and Clinical Safety (Figure 3F) cohorts to differentiate animal from human immunogenicity events.

Analysis of these subsets revealed that while animal immunogenicity was prevalent across both cohorts (36.4%), confirmed human immunogenicity was rare. *DiscoVerse* successfully located two positive human cases in the safety cohort and one in the efficacy cohort. Crucially, the system did not merely flag these molecules but provided the specific study context for these signals. This enabled the retrieval of latent immunogenicity data regarding specific molecules that were otherwise categorized broadly under safety or efficacy failures, providing experts with necessary evidence without requiring manual review of the entire historical archive.

## 6 Discussion and conclusion

This work shows that *DiscoVerse* can convert fragmented archival pharmaceutical documentation into traceable, actionable knowledge. Traditional research tools struggle with inconsistent formats and lost institutional memory. By decomposing scientific questions into aligned sub-tasks (preclinical, clinical, and strategic) and enforcing source-grounded synthesis via an orchestrating agent, *DiscoVerse* operationalizes a structured reasoning workflow that mirrors interdisciplinary scientific teams.

Across seven quantitative benchmarks (Q1–Q7), *DiscoVerse* achieved high recall (≥0.986) with moderate precision (0.71–0.91), a useful balance in safety-critical settings where missing true signals is costlier than reviewing extra candidates. Most false positives arose from contextual ambiguity rather than invention, indicating broad but evidence-linked retrieval. Qualitative evaluations on Q8 (Discontinuation Rationale) and Q9 (Multi-Phase Toxicity Evidence Integration) highlight the framework's ability to assemble disparate data into structured, auditable summaries.

A key feature of *DiscoVerse* is alignment with expert oversight. Rather than acting as an autonomous decision-maker, it automates exhaustive search and first-pass synthesis so scientists can adjudicate context and meaning. This design reduces hallucination risk and supports regulatory traceability, important in GLP/GVP environments. By linking source material through structured schemas, even negative or discontinued programs become analyzable, turning latent data into reusable evidence for reverse translation, hypothesis testing, target validation, and safety-margin estimation.

**Practical recommendations** Based on our experience developing and deploying *DiscoVerse*, we highlight several considerations for building LLM-based systems in pharmaceutical settings. (i) *Prioritize recall over precision in safety-critical retrieval*: missing a true safety signal is costlier than surfacing extra candidates for expert review; downstream human-in-the-loop verification can efficiently filter false positives. (ii) *Decompose complex queries into domain-aligned sub-tasks*: a single monolithic prompt cannot reliably handle queries spanning preclinical, clinical, and strategic dimensions; role-specialized agents with focused instructions reduce hallucination and improve interpretability. (iii) *Maintain source provenance at every step*: in regulated environments, an answer without a traceable evidence chain has limited utility regardless of its correctness. (iv) *Design for graceful degradation*: when documents are missing, poorly parsed, or ambiguous, the system should report uncertainty rather than fabricate plausible-sounding outputs. (v) *Invest in domain-expert evaluation*: automated metrics correlate poorly with scientific utility in pharmaceutical applications; expert adjudication, though resource-intensive, remains essential for validating system outputs.

Limitations include (i) moderate precision from context drift and incomplete metadata and (ii) need for manual final interpretation. Future work will focus on expert evaluation and broader evaluation on active programs and integration with *in-silico* modeling could further demonstrate translational value. Standardizing audit trails and quantifying uncertainty will support deployment in regulated contexts.

In conclusion, *DiscoVerse* shows how multi-agent LLM systems can serve as co-scientists in evidence-rich domains. By emphasizing grounding, explainability, and expert supervision rather than raw automation, it provides a blueprint for responsible AI integration in pharmaceutical research. When combined with institutional expertise, *DiscoVerse* converts historical archives into living knowledge, accelerating discovery, enhancing reproducibility, and ensuring past lessons inform future medicines.

## Data Availability

The datasets presented in this article are not readily available because all data related to this study is confidential internal data. Requests to access the datasets should be directed to the corresponding author.

## Footnotes

1 We computed token counts using OpenAI's tiktoken BPE tokenizer (https://github.com/openai/tiktoken) on text extracted from PDFs.
