# Supplementary material for: DiscoVerse: multi-agent pharmaceutical co-scientist for traceable drug discovery and reverse translation
Source: Front Artif Intell. 2026 Jun 18;9:1808378. doi: 10.3389/frai.2026.1808378 (PMC13323300; doi:10.3389/frai.2026.1808378)
Supplement: Supplementary file 1 [file Supplementary_File_1.pdf]

Appendix: DiscoVerse: Multi-Agent Pharmaceutical Co-Scientist for Traceable Drug Discovery and Reverse Translation

Xiaochen Zheng *
Predictive Modelling
F. Hoffmann-La Roche Ltd.
Basel, Switzerland

Alvaro Serra
Predictive Modelling
F. Hoffmann-La Roche Ltd.
Basel, Switzerland

Ilya Schneider Chernov
Predictive Modelling
F. Hoffmann-La Roche Ltd.
Basel, Switzerland

Maddalena Marchesi
Clinical Safety
F. Hoffmann-La Roche Ltd.
Basel, Switzerland

Eunice Musvasva
Translational Safety
F. Hoffmann-La Roche Ltd.
Basel, Switzerland

Tatyana Y. Doktorova
Predictive Modelling
F. Hoffmann-La Roche Ltd.
Basel, Switzerland

A Agent Workflow

When a user query is submitted to *DiscoVerse*, it undergoes a series of processing steps involving the agents described above. The end-to-end workflow is outlined below:

Query Classification and Decomposition (*Classification and Decomposition Agent* in Fig. ??) The classification agent analyzes the user's query and determine which knowledge domains it related to (preclinical, clinical, strategic, or a combination). The system then decomposes them into simpler but more detailed sub-queries using predefined decomposition rules. Simultaneously, the classification agent classifies the query into specific question types (such as toxicity, efficacy, or discontinuation reasons) based on categories defined collaboratively by project leaders and scientists. The *DiscoVerse* maintains a library of structured output schemas (templates) corresponding to each question type, and when a query spans multiple topics, the system combines relevant schemas into a single composite template to ensure the final answer captures all necessary facets.

Document Retrieval (*Search Agent* in Fig. ??) Once the sub-queries are defined and assigned to the appropriate domain agent, the next step is to retrieve relevant information from database. We employ a Hybrid document retriever that combines symbolic search with semantic search. The detailed methods are provided in Appendix B.

Relevance Reviewing (*Review Agent* in Fig. ??) The retrieval step typically yields a collection of document chunks that potentially contain the answer. Not all retrieved text will be truly relevant or high-quality, so each domain agent includes a review sub-process that filters these chunks. We utilize a reranker and an LLM-based relevance scoring mechanism: for each retrieved chunk, the agent's LLM is prompted with a question-answering relevance check. The reranker returns a relevance score which is mapped to a float value in $[0, 1]$ and LLM returns a judgment. Only chunks that both exceed a reranker score of 0.7 and have a relevance judgment are retained for analysis; the remainder are discarded to reduce noise.

Domain-Specific Evidence Extraction (*Research Agent* in Fig. ??) After filtering, each domain agent now has a curated set of relevant text passages for its sub-query. The next step is evidence extraction and synthesis. The agent composes a prompt that includes the user's sub-query and the collected text chunks (often truncated or summarized if they are long) and asks the LLM to generate a concise finding. This is effectively a summary or answer drawn from the evidence, focusing on the

*Corresponding author. xiaochen.zheng@roche.com

Preprint. Under review.

key details that address the question. The prompt templates for this step are tailored to each domain. We refer to these outputs as domain-specific findings. Throughout this process, the agents maintain the lineage of information so that evidence traceability is preserved (useful for later expert review, although in this paper we focus on the methodology rather than the user interface).

Multi-Agent Synthesis (*Supervisor Agent* in Fig. ??) The Supervisor (or orchestrator) agent waits for the domain agents to finish their analysis. Once all requested domain-specific findings are ready, the Supervisor integrates the results and assembles a coherent answer that covers each aspect. It may simply concatenate the findings under appropriate subheadings or, if needed, generate a brief narrative that connects them. The important point is that each piece of the answer is coming from a specialized analysis pipeline, ensuring depth in that area, and the Supervisor merges these pieces into a comprehensive response to the original query. The multi-agent coordination is implemented such that if one domain yields "no finding" (for instance, if nothing relevant was found in clinical data), the system can still return the findings from other domains along with a note that no information was available in that domain. This ensures graceful handling of negative or null results.

Consistent Structured Output (*Taxonomy Agent* in Fig. ??) Besides providing a long free-text report, *DiscoVerse* outputs the answer in a structured form. Each field in the schema corresponds to a specific piece of information, as shown later in Fig. A2 and A3. The content generated by the domain agents is mapped into these schema fields. If the query was multi-type and a composite schema was created, all relevant fields from each domain are included. In addition, the *Taxonomy Agents* operationalize a modular *schema library* co-designed with scientists and project leads, which contains predefined question types with concise descriptions, routing/classification rules, required evidence elements to extract, and structured output templates; a single user query may match multiple types, which are composed into a unified schema while preserving per-type provenance to support auditable synthesis.

Throughout this pipeline, by breaking the problem into smaller tasks handled by different agents, we reduce the cognitive load on any single LLM prompt and make the overall process more interpretable. The modular design also allowed us to incorporate fallback mechanisms: for example, within Classification Agent, if the LLM classification fails or is not available, a rule-based classifier steps in to ensure the query is still routed properly. Similarly, if the retriever finds too much data, the system can impose limits or stricter relevance thresholds to keep only the most salient information. We aim to continuously ingest and analyze historical research and development knowledge, and to provide scientists and decision-makers with an ever-ready, unbiased assistant to inform their next steps.

B Document Processing Pipeline and Retriever

B.1 PDF Parsing

We employed a Vision-Language Model (VLM)-based Optical Character Recognition (OCR) system, specifically olmOCR, for parsing PDF documents, as shown in Fig. A1. This approach preserves the hierarchical structure inherent in any kind of papers, including section and subsection organization, text formatting attributes (bold, italic, etc.), mathematical formulas and equations, tables and structured data, citation references.

B.2 Metadata Extraction

Following the parsing stage, we extract metadata from two sources: (1) the parsed markdown content, and (2) the raw database records, as shown in Fig. A1. Extracted metadata includes study title, drug identifier, temporal information, study stage, and relevant keywords. This metadata is then embedded alongside the corresponding text chunks during the embedding process, enriching the semantic representation of each chunk with structured contextual information.

B.3 Text Chunking

Documents were segmented into chunks of 512 words with a 64-word overlap between consecutive chunks. The overlap preserves contextual continuity across chunk boundaries.

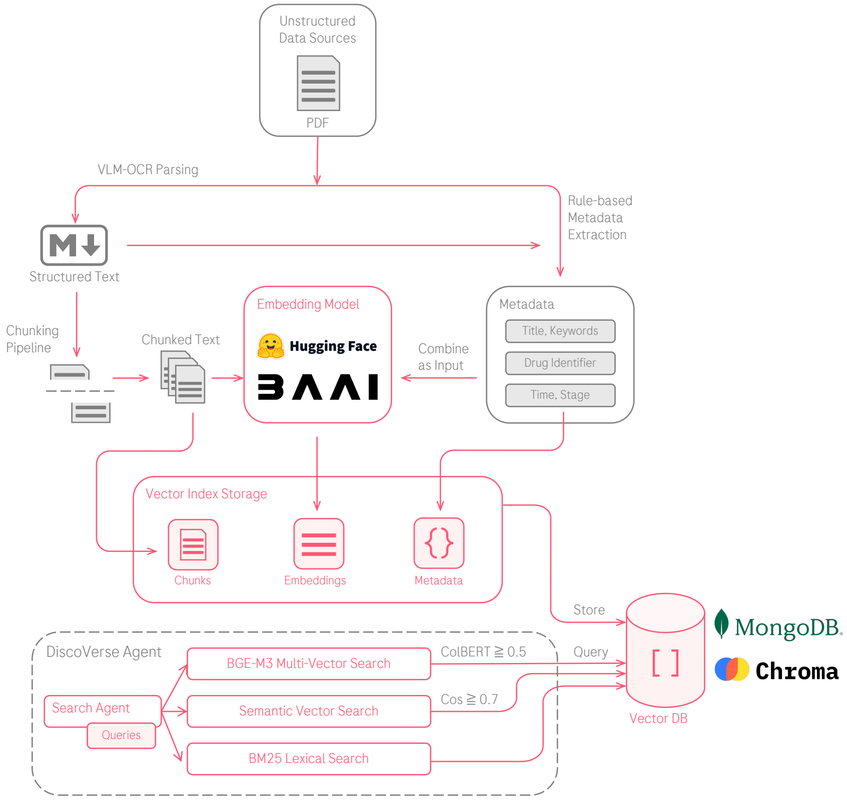

Figure A1: The illustrative overview of document processing and retriever in *DiscoVerse*.

To maintain semantic coherence, our chunking implementation respects section boundaries. Chunks do not break in the middle of logical sections. This section-aware approach ensures that retrieved passages maintain their original contextual structure.

B.3.1 Embedding Models

We utilized two multilingual embedding models to capture semantic representations of text chunks, as described in Fig. A1:

(1) intfloat/multilingual-e5-large-instruct: A multilingual embedding model with instruction-following capabilities, enabling task-specific encoding through natural language instructions.

(2) BGE-M3: A hybrid embedding model supporting dense retrieval, lexical matching, and multi-vector (ColBERT-style) representations within a unified framework.

B.4 Hybrid Retrieval System

We implemented a hybrid retrieval system combining three retrieval mechanisms:

(1) Semantic Vector Search: Dense retrieval using the multilingual-e5-large-instruct embeddings with a similarity threshold of 0.7

(2) BGE-M3 Multi-Vector Search: ColBERT-style late interaction retrieval with a minimum ColBERT score threshold of 0.5

(3) BM25 Lexical Search: Keyword-based retrieval for exact term matching

Results from all three retrievers are merged and deduplicated to produce the final candidate set. This hybrid approach combines semantic understanding with lexical precision.

The retrieval infrastructure uses two storage backends: ChromaDB (an open-source vector database) for vector similarity search operations and MongoDB (deployed on-premises) for document storage and metadata management.

C Expert Evaluation Design

Evaluating LLMs in pharmaceutical applications requires particular care given the safety-critical nature of drug development decisions. A key concern is hallucination, which poses significant risks when LLMs are deployed to extract clinical data or support regulatory submissions (Hakim et al., 2025). However, standard evaluation approaches using automated metrics like ROUGE (Lin, 2004) and BLEU (Papineni et al., 2002) are insufficient for this domain, as they correlate poorly (sometimes negatively) with how medical experts judge accuracy (Wang et al., 2023; Ben Abacha et al., 2023; Nguyen et al., 2024; Fraile Navarro et al., 2025). Expert evaluation becomes essential and crucial for medical and drug development applications (Agrawal et al., 2025; Kim et al., 2025; Schilling-Wilhelmi et al., 2025; Zhu et al., 2025; Rieff et al., 2025; Gu et al., 2025). We therefore use expert evaluation of source-linked outputs; while this increases review burden, it better captures scientific utility. Our evaluation protocol, centered on expert judgment, distinguishes *DiscoVerse*, and to our knowledge no prior research has systematically evaluated agentic systems for drug discovery workflows on real pharmaceutical data, particularly for reverse translation. Additionally, *DiscoVerse* is the first platform with authorized access to confidential data generated during the drug development process. Such access is essential for the nature of our research, which requires comprehensive and context-rich information spanning the full drug development lifecycle.

To demonstrate the value *DiscoVerse* brings to pharmaceutical decision-making, we design the evaluation targeting the core challenge faced by research and development teams: *extracting discrete, decision-critical information from decades of unstructured, heterogeneous documentation*. This evaluation directly mirrors real-world scenarios where scientists must synthesize evidence across discontinued programs to inform current development decisions. This task traditionally requiring weeks of manual review or remaining unaddressed due to resource constraints.

To keep the study rigorous yet tractable, we selected seven representative (Q1 - Q7), decision-critical queries for quantitative expert-in-the-loop evaluation. For each query, *DiscoVerse* retrieved source-linked document chunks and generated answers that pharmaceutical scientists independently adjudicated against the original sources, labeling each response as True Positive (TP), True Negative (TN), False Positive (FP), or False Negative (FN) according to prespecified criteria for factual accuracy and contextual completeness (Gartlehner et al., 2024). We choose **accuracy**, **precision**, **recall**, **specificity**, **F1-score** to evaluate the performance. Detailed evaluation criteria and per-query rubrics are provided in Appendix D and E.

The remaining two queries (Q8 and Q9) were not scored quantitatively across all drug programs because (i) the original project leads have left, and the underlying “real reason” is not written explicitly in our decades-spanning repository; it is latent, spread across reports, raw data, figures, and protocol amendments, making consistent program-wide scoring infeasible; and (ii) some key archival documents are missing or incomplete. This is exactly why we built *DiscoVerse*: role-specialized agents retrieve, link, and synthesize dispersed evidence to surface these otherwise hidden answers. Even though the answers we provide for Q8 and Q9 cannot be evaluated quantitatively for the two reasons mentioned above, they hold significant value in offering an overview of the information found and available in the documents associated with the particular Roche molecule. These findings also provide valuable insights for further understanding and analysis.

D Evaluation Design for Benchmark Questions

Each LLM output was manually reviewed against its source document and classified into one of four categories: True Positive (TP), True Negative (TN), False Positive (FP), or False Negative (FN). The evaluation criteria were tailored to each of the seven queries for all 180 drugs as detailed below and summarized in Tab. A1.

D.1 Query 1: First-in-Human (FIH) Dose

Query: "What was the first in human dose for (molecule)?"

Positive Case: The document explicitly states the FIH dose.

Negative Case: The document discusses the molecule but does not mention the FIH dose.

	TP	TN	FP	FN
**Q1: FIH Dose**	Value Identified ✓ AND Context (if given) ✓	Correctly states info is absent ✓	Incorrect Value ✗ OR Preclinical Value ✗ OR Hallucination ✗	Fails to find present value ✗
**Q2: RoA**	Correct Route ✓ AND Context (if given) ✓	Correctly states info is absent ✓	Incorrect Route ✗ OR Preclinical Route ✗	Fails to find present RoA ✗
**Q3: Highest Dose**	Highest Value Identified ✓ AND Correct Phase ✓	Correctly states info is absent ✓	Incorrect Value ✗ OR Incorrect Phase ✗ OR Not Highest Value ✗	Fails to find present value ✗
**Q4: Dose w/ SAEs**	Correct Dose ✓ AND Correct Link to SAE ✓	Correctly states info is absent ✓	Incorrect Dose ✗ OR Incorrect Link to SAE ✗ OR Confuses AE/SAE ✗	Fails to connect present dose–SAE link ✗
**Q5: Efficacious Dose**	Correct Dose ✓ AND Context (if given) ✓	Correctly states info is absent ✓	Incorrect Dose ✗ OR Misrepresents Efficacy ✗	Fails to find present efficacy info ✗
**Q6: Regimen**	All components correct (Dose, Freq., Duration) ✓	Correctly states info is absent ✓	One or more components incorrect ✗	Fails to synthesize present regimen details ✗
**Q7: Safety Margin**	Synthesizes correct preclinical AND/OR clinical data ✓	Correctly states info is absent ✓	Uses incorrect values (e.g., toxic dose) for synthesis ✗	Fails to connect present data to describe margin ✗

Table A1: Definition of TP, TN, FP, FN for seven benchmark questions respectively.

Classification Criteria:

- **True Positive (TP):** The LLM correctly extracts the FIH dose and its accompanying context is factually correct. For example, extracting "The FIH was 5 mg/kg". If context is given (e.g. the correct study population and trial phase) it also has to be correct.
- **False Positive (FP):** The LLM extracts an incorrect dose, such as a preclinical dose from an animal study, or hallucinates a value not present in the text.
- **False Negative (FN):** The LLM fails to find the FIH dose even though it is stated in the document.
- **True Negative (TN):** The LLM correctly reports that the information is not available in the provided text.

D.2 Query 2: Route of Administration (RoA)

Query: "What was the route of administration in humans for drug X?"

Positive Case: The document mentions how the drug was administered to humans (e.g., oral, intravenous).

Negative Case: The route of administration is not mentioned.

Classification Criteria:

- **True Positive (TP):** The LLM correctly identifies the route of administration.
- **False Positive (FP):** The LLM states the wrong route or confuses a preclinical (animal) route with the human route.
- **False Negative (FN):** The LLM fails to find the RoA even though it is stated.
- **True Negative (TN):** The LLM correctly reports that the RoA is not mentioned.

D.3 Query 3: Highest Dose in Phase I(MAD)/II

Query: "What was the highest clinical dose in Phase I(MAD)/II for (molecule)?"

Positive Case: The document contains dose information for Phase I Multiple Ascending Dose (MAD) and/or Phase II studies.

Negative Case: The document does not contain this specific dose information.

Classification Criteria:

- **True Positive (TP):** The LLM correctly identifies the single highest dose administered across all relevant study phases mentioned.
- **False Positive (FP):** The LLM extracts a dose from an incorrect phase (e.g., Phase III), fails to identify the maximum value among several options, or incorrectly identifies a dose as being from a MAD study.
- **False Negative (FN):** The LLM reports that the information is not found despite it being present.
- **True Negative (TN):** The LLM correctly determines that no dose information for Phase I(MAD) or Phase II is available.

D.4 Query 4: Highest Dose with Severe Adverse Events (SAEs)

Query: "What was the highest clinical dose at which there were severe adverse events for drug (molecule)?"

Positive Case: The document explicitly links a dose level to the occurrence of SAEs, Dose-Limiting Toxicities (DLTs), or Grade 3+ adverse events.

Negative Case: The document reports no SAEs or does not link them to a specific dose.

Classification Criteria:

- **True Positive (TP):** The LLM correctly extracts the dose that is explicitly associated with an SAE or DLT.
- **False Positive (FP):** The LLM incorrectly associates a dose with an unrelated SAE or confuses a mild adverse event with a severe one. This represents a critical failure in relational extraction.
- **False Negative (FN):** The LLM fails to connect a dose and an SAE even when the relationship is clearly stated.
- **True Negative (TN):** The LLM correctly reports that no dose was explicitly linked to SAEs.

D.5 Query 5: Efficacious Dose

Query: "What was the efficacious dose in the clinic?"

Positive Case: The document discusses clinical efficacy, pharmacodynamic markers, or therapeutic response at specific doses.

Negative Case: The document only discusses safety and/or pharmacokinetics, not efficacy.

Classification Criteria:

- **True Positive (TP):** The LLM correctly extracts the efficacious dose(s) and accurately summarizes the associated outcomes and patient populations.
- **False Positive (FP):** The LLM extracts a dose but misrepresents its efficacy (e.g., claims a dose was effective when the trial failed to show a benefit).
- **False Negative (FN):** The LLM fails to find the efficacy information despite it being present in the text.
- **True Negative (TN):** The LLM correctly reports that no efficacy data is available.

D.6 Query 6: Treatment Regimen

Query: "What was the treatment regimen for drug X in humans?"

Positive Case: The document describes the complete dosing schedule (dose level, frequency, duration).

Negative Case: The regimen details are not described.

Classification Criteria:

- **True Positive (TP):** The LLM correctly synthesizes the dose level(s), frequency, duration, and patient population into a coherent summary.
- **False Positive (FP):** The LLM gets one or more elements of the regimen wrong (e.g., mistakes a single-dose study for a multiple-dose study).
- **False Negative (FN):** The LLM fails to assemble the regimen details from the text.
- **True Negative (TN):** The LLM correctly reports that the regimen is not detailed.

D.7 Query 7: Margin of Safety

Query: "What do we know about the Margin of Safety of drug X in the first IB?"

Positive Case: The document contains preclinical toxicology data (e.g., NOAEL) and the clinical starting dose.

Negative Case: This information is not present to calculate or assess the safety margin.

Classification Criteria:

- **True Positive (TP):** The LLM correctly synthesizes preclinical safety data and clinical dose data to accurately describe the safety margin.
- **False Positive (FP):** The LLM uses incorrect numbers (e.g., a toxic dose instead of a no-effect dose), leading to a miscalculation or misrepresentation of the safety margin.
- **False Negative (FN):** The necessary data is present, but the LLM fails to connect them to describe the safety margin.
- **True Negative (TN):** The LLM correctly reports that the data needed to assess the safety margin is absent.

E Overall Evaluation Metrics

The following metrics were calculated for each of the seven benchmark questions:

Accuracy: The overall proportion of correct predictions, calculated as

$$\text{Accuracy} = \frac{TP + TN}{TP + TN + FP + FN} \tag{1}$$

Precision (Positive Predictive Value): The proportion of positive predictions that were factually correct, calculated as

$$\text{Precision} = \frac{TP}{TP + FP} \tag{2}$$

This metric is a direct measure of the system's factuality and its tendency to avoid hallucinations or contextually incorrect extractions.

Recall (Sensitivity): The proportion of all actual positive cases that were correctly identified by the system, calculated as

$$\text{Recall} = \frac{TP}{TP + FN} \tag{3}$$

This metric measures the agent's completeness and its ability to successfully find the "needle" when it is present.

Specificity: The proportion of all actual negative cases that were correctly identified, calculated as

$$\text{Specificity} = \frac{TN}{TN + FP} \tag{4}$$

This measures the agent's ability to correctly recognize the absence of information.

F1-Score: The harmonic mean of Precision and Recall, calculated as

$$\text{F1-Score} = 2 \times \frac{\text{Precision} \times \text{Recall}}{\text{Precision} + \text{Recall}} \tag{5}$$

It provides a single, balanced score that accounts for both factuality and completeness.

F Qualitative Evaluation Findings

Queries Q8 (Discontinuation Rationale) and Q9 (Multi-Phase Toxicity Evidence Integration) were evaluated qualitatively rather than through the quantitative TP/FP/TN/FN framework applied to Q1-Q7. In parallel, we continue collaborating with project leads to retrieve missing records and refine our approach through iterative feedback on prompt formulation and task decomposition. While quantitative evaluation of these responses is not feasible for the reasons noted above, they nonetheless provide a valuable synthesis of the available evidence across documents linked to each Roche molecule. This synthesis offers meaningful perspectives that enhance contextual understanding and inform subsequent methodological enhancements.

Discontinuation Rationale (Q8) Q8 presents unique evaluation challenges because discontinuation of a drug candidate is typically reported under one or two official categories (e.g., clinical safety, preclinical safety, or clinical efficacy). In reality, however, discontinuation decisions are often multifactorial and result from the interaction of several contributing elements rather than a single definitive cause. A program may be terminated due to a combination of preclinical or clinical issues (e.g., safety, efficacy, or bioavailability), outcomes of clinical futility analyses, strategic portfolio reprioritization, and resource limitations. The relative influence of these factors reflects an institutional decision-making context that can be definitively confirmed only by the original project leads. Consequently, although the official documentation or labeling may list a single primary reason for discontinuation, such classifications rarely capture the full complexity of the underlying decision process.

As shown in Fig. A2, this example illustrates how DiscoVerse can accurately identify that the reason for discontinuation originates in the preclinical phase, rather than being driven by clinical or other strategic factors.

Multi-Phase Toxicity Evidence Integration (Q9) Q9 presents analogous evaluation challenges: toxicity information is fragmented across the entire drug development lifecycle, spanning preclinical and clinical phases. Crucially, many of these documents contain evolving, preliminary, or context-dependent findings rather than definitive conclusions about whether a compound exhibits organ-specific toxicity (e.g., hematotoxicity). As visualized in Fig. A3, *DiscoVerse* decomposes the user query into distinct sub-tasks that separately interrogate preclinical and clinical evidence sources. The preclinical reasoning chain aggregates findings from 14-day and 28-day toxicity studies in rats and dogs, along with in vitro assays. Simultaneously, the clinical reasoning chain confirms that no human data are available, thereby preventing spurious claims of toxicity. The structured output builder consolidates these threads into a cross-species toxicity matrix summarizing the evidence base.

This example highlights the model's ability to perform multi-phase evidence integration: it synthesizes distributed, heterogeneous data sources and reconciles apparently conflicting or incomplete evidence streams into a coherent structured summary.

References

M. Agrawal, I. Y. Chen, F. Gulamali, and S. Joshi. The evaluation illusion of large language models in medicine. *npj Digital Medicine*, 8(1):600, 2025.

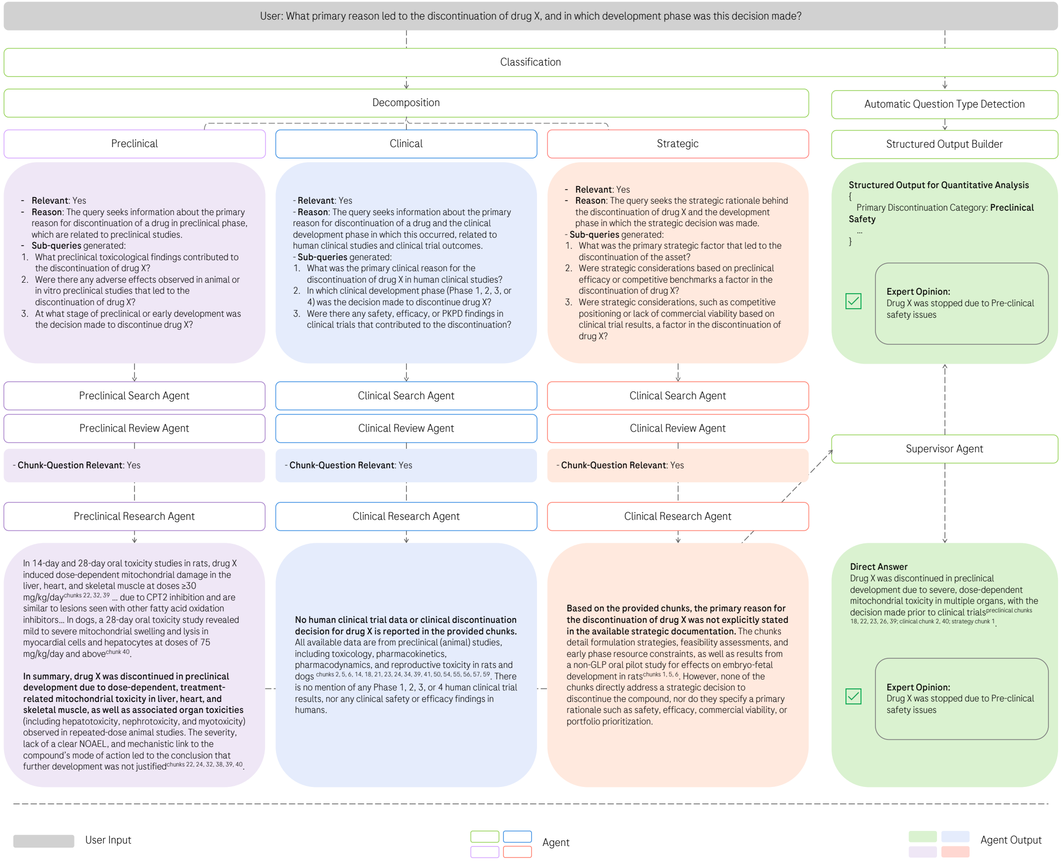

Figure A2: The illustrative output of Q8 (Discontinuation Rationale).

A. Ben Abacha, W.-w. Yim, G. Michalopoulos, and T. Lin. An investigation of evaluation methods in automatic medical note generation. In *Findings of the Association for Computational Linguistics: ACL 2023*. Association for Computational Linguistics, 2023.

D. Fraile Navarro, E. Coiera, T. W. Hambly, Z. Triplett, N. Asif, A. Susanto, A. Chowdhury, A. Azcoaga Lorenzo, M. Dras, and S. Berkovsky. Expert evaluation of large language models for clinical dialogue summarization. *Scientific reports*, 15(1):1195, 2025.

G. Gartlehner, L. Kahwati, B. Nussbaumer-Streit, K. Crotty, R. Hilscher, S. Kugley, M. Viswanathan, I. Thomas, A. Konet, G. Booth, et al. From promise to practice: challenges and pitfalls in the evaluation of large language models for data extraction in evidence synthesis. *BMJ Evidence-Based Medicine*, 2024.

L. Gu, Y. Zhu, H. Sang, Z. Wang, D. Sui, W. Tang, E. Harrison, J. Gao, L. Yu, and L. Ma. Medagentaudit: Diagnosing and quantifying collaborative failure modes in medical multi-agent systems. *arXiv preprint arXiv:2510.10185*, 2025.

J. B. Hakim, J. L. Painter, D. Ramcharran, V. Kara, G. Powell, P. Sobczak, C. Sato, A. Bate, and A. Beam. The need for guardrails with large language models in pharmacovigilance and other medical safety critical settings. *Scientific Reports*, 15(1):27886, 2025.

H. Kim, J. Sohn, A. Gilson, N. Cochran-Caggiano, S. Applebaum, H. Jin, S. Park, Y. Park, J. Park, S. Choi, B. A. H. Contreras, T. Huang, J. Yun, E. F. Wei, R. Jiang, L. Colucci, E. Lai, A. Dave, T. Guo, M. B. Singer, Y. Koo, R. A. Adelman, J. Zou, A. Taylor, A. Cohan, H. Xu, and Q. Chen. Rethinking retrieval-augmented generation for medicine: A large-scale, systematic expert evaluation and practical insights. *arXiv preprint arXiv:2511.06738*, 2025. URL https://arxiv.org/abs/2511.06738.

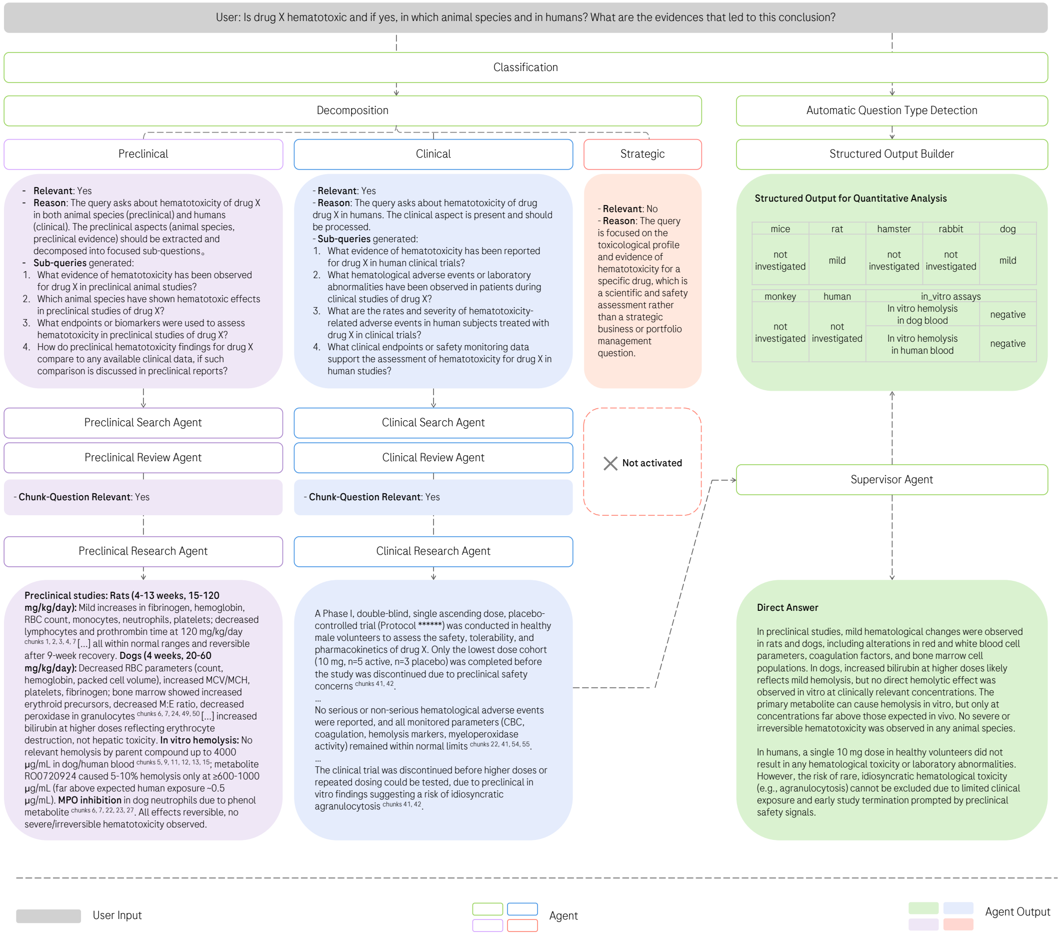

Figure A3: The illustrative output of Q9 (Multi-Phase Toxicity Evidence Integration).

C.-Y. Lin. ROUGE: A package for automatic evaluation of summaries. In *Text Summarization Branches Out*. Association for Computational Linguistics, 2004.

H. Nguyen, H. Chen, L. Pobbathi, and J. Ding. A comparative study of quality evaluation methods for text summarization. *arXiv preprint arXiv:2407.00747*, 2024.

K. Papineni, S. Roukos, T. Ward, and W.-J. Zhu. Bleu: a method for automatic evaluation of machine translation. In *Proceedings of the 40th Annual Meeting of the Association for Computational Linguistics*. Association for Computational Linguistics, 2002.

M. Rieff, M. Varma, O. Rabow, S. Adithan, J. Kim, K. Chang, H. Lee, N. Rohatgi, C. Bluethgen, M. S. Muneer, J.-B. Delbrouck, and M. Moor. SMMILE: An expert-driven benchmark for multimodal medical in-context learning. In *The Thirty-ninth Annual Conference on Neural Information Processing Systems (NeurIPS) Datasets and Benchmarks Track*, 2025. URL https://openreview.net/forum?id=ZONgHRsAbl.

M. Schilling-Wilhelmi, M. Ríos-García, S. Shabih, M. V. Gil, S. Miret, C. T. Koch, J. A. Márquez, and K. M. Jablonka. From text to insight: large language models for chemical data extraction. *Chemical Society Reviews*, 2025.

L. L. Wang, Y. Otmakhova, J. DeYoung, T. H. Truong, B. Kuehl, E. Bransom, and B. Wallace. Automated metrics for medical multi-document summarization disagree with human evaluations. In *Proceedings of the 61st Annual Meeting of the Association for Computational Linguistics*. Association for Computational Linguistics, 2023.

Y. Zhu, Z. He, H. Hu, X. Zheng, X. Zhang, Z. Wang, J. Gao, L. Ma, and L. Yu. MedAgentBoard: Benchmarking multi-agent collaboration with conventional methods for diverse medical tasks. In *The Thirty-ninth Annual Conference on Neural Information Processing Systems (NeurIPS) Datasets and Benchmarks Track*, 2025. URL https://openreview.net/forum?id=BPpG4qQaNj.
